# Supplementary material for: Reconciling Mining with the Conservation of Cave Biodiversity: A Quantitative Baseline to Help Establish Conservation Priorities
Source: PLoS One. 2016 Dec 20;11(12):e0168348. doi: 10.1371/journal.pone.0168348 (PMC5173368; doi:10.1371/journal.pone.0168348)
Supplement: S1 Dataset — (ZIP) [file pone.0168348.s002.zip › Taxa/Serra Norte/SN_2013/N4E/Lista N4E-66.pdf]

CAVIDADE N4E-0066

Classe	Ordem	Fam/Outros	Gên/Outros	Espécie	S-U	U-S	Zon.
Arachnida	Acari	Gamasida		jovem		X	E
Arachnida	Acari	Ixodidae	*Amblyomma*	sp.1		X	E
Arachnida	Araneae	Ctenidae		jovem		1	E
Arachnida	Araneae	Filistatidae		sp.1	X	X	E
Arachnida	Araneae	Oonopidae	aff. *Triaeris*	sp.2		X	E
Arachnida	Araneae	Pholcidae	*Mesabolivar*	jovem	X	X	E
Arachnida	Araneae	Salticidae		sp.2		X	E
Arachnida	Araneae	Scytodidae	*Scytodes*	*eleonorae*	5	4	E
Arachnida	Araneae	Tetragnathidae		jovem		X	E
Arachnida	Araneae	Theridiidae	*Dipoena*	sp.4	X		E
Arachnida	Araneae	Theridiidae	*Theridion*	sp.1	X	X	E
Arachnida	Araneae	Theridiosomatidae	*Plato*	sp.1	X	X	E
Arachnida	Opiliones	Escadabiidae		sp.2	X		E
Arachnida	Opiliones	Stygnidae	*Paraphareus*	sp.1		X	E
Chilopoda	Geophilomorpha	Geophilidae		sp.1		1	E
Diplopoda	Polydesmida	Pyrgodesmidae		sp.1		X	E
Diplopoda	Spirostreptida	Pseudonannolenidae	*Pseudonannolene*	jovem	1		E
Entognatha	Collembola	Paronellidae		sp.1		X	E
Insecta	Blattodea	Blaberidae		jovem	1		E
Insecta	Blattodea	Blattidae		sp.5	7		E
Insecta	Blattodea	Polyphagidae		jovem		4	E
Insecta	Coleoptera	Chrysomelidae		sp.15		X	E
Insecta	Coleoptera			jovem	X	X	E
Insecta	Diptera	Culicidae	*Culex*	sp.		X	E
Insecta	Heteroptera	Cydnidae		sp.	X		E
Insecta	Heteroptera	Reduviidae	Emesinae	sp.		4	E
Insecta	Heteroptera	Reduviidae	Reduviinae	sp.	5	3	E
Insecta	Homoptera	Cixiidae		jovem		X	E
Insecta	Homoptera	Membracidae		sp.1	X		E
Insecta	Hymenoptera	Formicidae	*Camponotus*	sp.1	X	X	E
Insecta	Hymenoptera	Formicidae	*Camponotus*	sp.2		X	E
Insecta	Hymenoptera	Formicidae	Myrmicinae	sp.1		X	E
Insecta	Hymenoptera	Formicidae	*Pheidole*	sp.3		X	E
Insecta	Hymenoptera	Formicidae	*Solenopsis*	sp.1	X		E
Insecta	Isoptera	Termitidae	*Nasutitermes*	sp.	X	X	E
Insecta	Lepidoptera	Noctuoidea		sp.5		2	E
Insecta	Lepidoptera	Tineoidea		sp.1	X		E
Insecta	Lepidoptera	Tineoidea		sp.2	X	X	E
Insecta	Neuroptera	Myrmeleontidae		jovem		X	E
Insecta	Orthoptera	Phalangopsidae	*Paraclodes*	sp.	1	10	E
Insecta	Plecoptera			jovem		1	E
Insecta	Psocoptera	Psyllipsocidae	*Psocathropos*	sp.1	X		E
Insecta	Psocoptera	Psyllipsocidae	*Psyllipsocus*	sp.3	X		E
Insecta	Psocoptera			jovem		X	E
Insecta	Thysanura	Nicoletiidae		sp.1	X	X	E
Malacostraca	Decapoda	Pseudothelphusidae		sp.1		1	E
Malacostraca	Isopoda	Scleropactidae		sp.1		X	E
Symphyla		Scutigerellidae		sp.		X	E
Amphibia	Anura	Strabomantidae	*Pristimantis*	cf. *fenestratus*	2	1	E

Mammalia	Chiroptera	Emballonuridae	*Peropteryx*	*kappleri*	6	8	E
